# Kava as a Clinical Nutrient: Promises and Challenges

**DOI:** 10.3390/nu12103044

**Published:** 2020-10-05

**Authors:** Tengfei Bian, Pedro Corral, Yuzhi Wang, Jordy Botello, Rick Kingston, Tyler Daniels, Ramzi G. Salloum, Edward Johnston, Zhiguang Huo, Junxuan Lu, Andrew C. Liu, Chengguo Xing

**Affiliations:** 1 Department of Medicinal Chemistry, College of Pharmacy, University of Florida, Gainesville, FL 32610, USA; tbian@cop.ufl.edu (T.B.); pcorral19@cop.ufl.edu (P.C.); wangyuzhi@cop.ufl.edu (Y.W.); jordybot@hotmail.com (J.B.); 2 College of Pharmacy, University of Minnesota, Minneapolis, MN 55455, USA; kingston@umn.edu; 3 Thorne Research Inc., Industrial Road, 620 Omni Dr, Summerville, SC 29483, USA; TDaniels@thorne.com; 4 Department of Health Outcome & Biomedical Informatics, College of Medicine, University of Florida, Gainesville, FL 32610, USA; rsalloum@ufl.edu; 5 The Association for Hawaiian Awa (kava), Pepe’ekeo, HI 96783, USA; aliapoint@gmail.com; 6 Department of Biostatistics, College of Public Health & Health Professions, College of Medicine, University of Florida, Gainesville, FL 32610, USA; zhuo@ufl.edu; 7 Department of Pharmacology, Penn State University College of Medicine, Hershey, PA 17033, USA; junxuanlu@pennstatehealth.psu.edu; 8 Department of Physiology and Functional Genomics, College of Medicine, University of Florida, Gainesville, FL 32610, USA; andrew.liu@ufl.edu

**Keywords:** kava, kavalactone, cultivars, quality control, quality assurance, stress, anxiety, cancer, hepatotoxicity, inflammation

## Abstract

Kava beverages are typically prepared from the root of Piper methysticum. They have been consumed among Pacific Islanders for centuries. Kava extract preparations were once used as herbal drugs to treat anxiety in Europe. Kava is also marketed as a dietary supplement in the U.S. and is gaining popularity as a recreational drink in Western countries. Recent studies suggest that kava and its key phytochemicals have anti-inflammatory and anticancer effects, in addition to the well-documented neurological benefits. While its beneficial effects are widely recognized, rare hepatotoxicity had been associated with use of certain kava preparations, but there are no validations nor consistent mechanisms. Major challenges lie in the diversity of kava products and the lack of standardization, which has produced an unmet need for quality initiatives. This review aims to provide the scientific community and consumers, as well as regulatory agencies, with a broad overview on kava use and its related research. We first provide a historical background for its different uses and then discuss the current state of the research, including its chemical composition, possible mechanisms of action, and its therapeutic potential in treating inflammatory and neurological conditions, as well as cancer. We then discuss the challenges associated with kava use and research, focusing on the need for the detailed characterization of kava components and associated risks such as its reported hepatotoxicity. Lastly, given its growing popularity in clinical and recreational use, we emphasize the urgent need for quality control and quality assurance of kava products, pharmacokinetics, absorption, distribution, metabolism, excretion, and foundational pharmacology. These are essential in order to inform research into the molecular targets, cellular mechanisms, and creative use of early stage human clinical trials for designer kava modalities to inform and guide the design and execution of future randomized placebo controlled trials to maximize kava’s clinical efficacy and to minimize its risks.

## 1. Introduction

**Figure 1:**
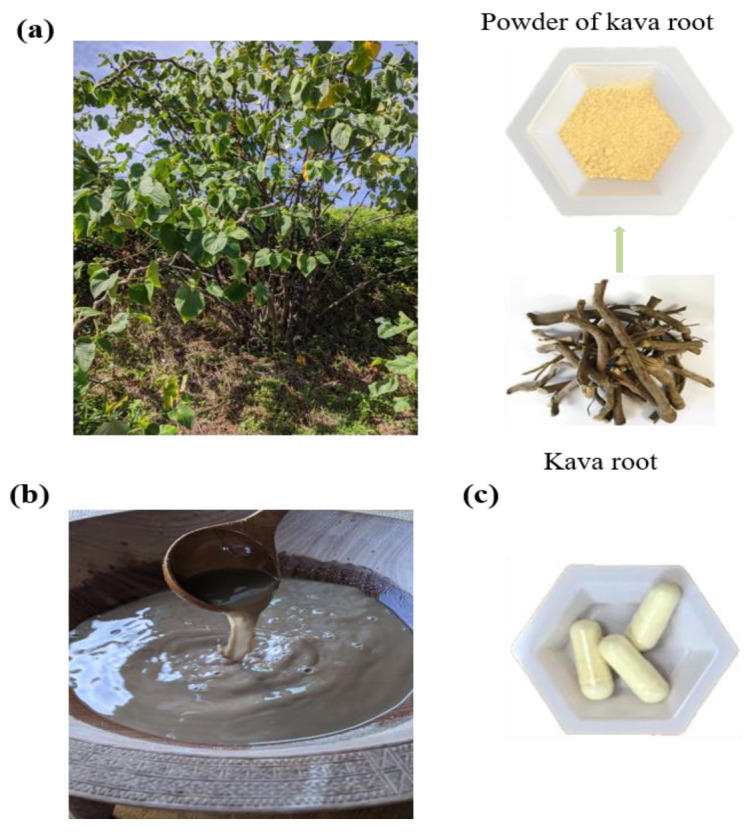
(**a**) The whole kava plant, kava roots, and the powder from kava roots; (**b**) traditional preparation; and (**c**) a commercial dietary supplement form of kava product.

Kava, also known as kava kava, ‘Awa, or ‘awa, is a type of perennial shrub that belongs to the pepper family, known as Piperaceae [1]. *Piper methysticum* is its botanical name, which derives from the Latin “methysticum”. In the local language and culture, the word “kava” is used to denote something “bitter”. Kava is native to Oceania, with important cultural and historical significance. It has been grown throughout the regions of Micronesia, Polynesia and Melanesia for its relaxant and medicinal effects as a pain reliever, muscle relaxant, and as a remedy for anxiety, nervousness and insomnia. Kava has been domesticated in these regions for thousands of years [2] (Figure 1a). Female flowers of kava are scarce and fruit pollination is not particularly productive. Kava is cultivated by propagation from stem cuttings [3]. It has over one hundred different chemotypes and cultivars. A set of lactones are abundant and present almost exclusively in kava, thus named kavalactones. These kavalactones are also believed to be responsible for the health benefits of traditional kava preparations [4]. Their fingerprints have also been used to distinguish different kava cultivars. The sum of the six major kavalactones has been used to standardize different kava products. Kava is consumed in the traditional form as a beverage, in a more merchandized form as either an anxiolytic agent or a dietary supplement, or more recently as a recreational drink served in kava bars.

**Figure 2:**
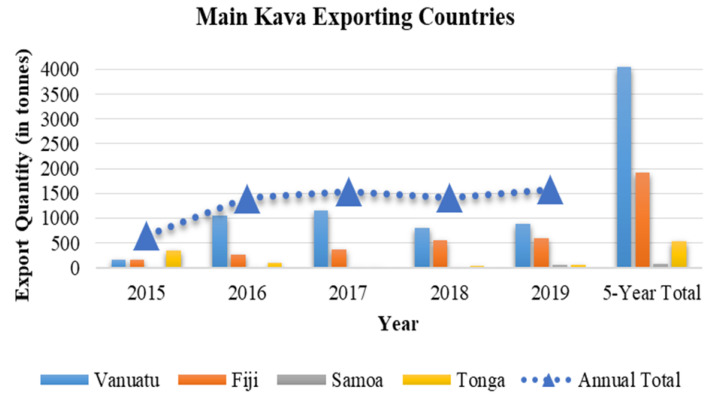
Kava exports from the main exporting countries between 2015 and 2019.

Based on export data from the main producing regions, kava’s demand and exposure has increased significantly in the past few years [5,6,7,8,9,10] (Figure 2). Consistency and quality, however, remain important issues that need to be better controlled, because they are critical to the efficacy and safety of kava products, as reviewed later. Whereas the neurological benefits of proper kava use appear to be undeniable, particularly in reducing stress and anxiety, there is growing evidence that its consumption could be associated with low incidence of cancer. Furthermore, anti-inflammatory activities have been reported for kava and its compounds, suggesting their potential for the treatment of inflammatory diseases. The mechanisms and responsible ingredients for these functions require further exploration and validation. This review, therefore, focuses on kava’s chemical composition and diversity, its anti-inflammatory potential, its neurological effects, its anti-cancer properties, and, finally, issues relating to its safety.

## 2. Kava and Its Diversity

### 2.1. Different Forms of Kava

The consumption of traditional forms of kava has a long history, with the product considered sacred in Pacific cultures and being served during significant events, such as when welcoming guests, celebrating childbirth, during marriages, as well as at funerals. Traditional kava use in these cultures has extended beyond important occasions in recent history, with most of the local residents drinking a cup before a meal. Kava can also act as an inebriating beverage, inducing physiological and psychological relaxation. This plant has become an essential part of Pacific Island societies, particularly in some of the major kava producing regions with popular traditional kava use, including Vanuatu, Fiji, Tonga, and Samoa [11,12]. Traditional kava is typically prepared from the root of the plant (fresh or dry) although some regions use peelings or stems as well. Typically, the dried root is ground into a powder, which is then mixed with ambient temperature water or coconut milk, and filtered through a cloth into a suspension (Figure 1b) [13,14]. The estimated doses of traditional kava use (Table 1) vary substantially across various regions, ranging from 750 to 8000 mg kavalactones/day [15,16,17,18]. Traditional kava use has been ubiquitously accepted to be safe in these regions, aligned with the recommendation from the World Health Organization (WHO) in its 2016 report [15].

In addition to its traditional use, kava has been used as a herbal drug for the treatment of anxiety, for which kavalactones have been documented as the responsible ingredients [4]. A number of clinical trials showed that kava was efficacious within the daily dose range of 20–300 mg kavalactones, particularly for mild and moderate anxiety [14,19,20,21,22,23,24,25]. Because of the potential limitations of the traditional preparations, such as low yield and high cost, non-traditional forms of kava have been developed. One common method of preparation for anxiolytic kava is ethanol or acetone extraction and the solvent-free extract is packaged into the final product in the form of tablets and capsules [26]. Due to the potential risk of hepatotoxicity from these products, Germany banned kava’s clinical anxiolytic use in 2002. Although the ban was lifted in 2014 [27], more clinical data are required before its reentry as a clinical anxiolytic agent.

Meanwhile, kava has been marketed for several decades in the form of dietary supplements in the U.S. to relieve stress, improve sleep and memory, and regulate mood [28]. The recommended daily dose for an adult is typically one to three capsules with a daily dose of 60–250 mg kavalactones for one or two months [15]. Some kava dietary supplements are prepared from the ethanolic extract in capsule form. Kava alcohol tinctures are also available as dietary supplements. This is a questionable practice given the potential hepatic interactions between kava and ethanol, which are discussed later in this review. In some products, capsules have been directly filled with kava powder and marketed as dietary supplements; it remains to be determined whether this is a good practice.

Kava has also been consumed as a social drink. The history of kava bars originated in New Caledonia, where young people wanted to have a place to meet and relax without drinking alcohol. Kava bars later expanded to Hawaii and have sprung up on islands in the Pacific and in some Western nations in recent years [29]. In the United States, for example, the number of kava bars increased by approximately 30% from 2012 to 2017 [16,30,31].

### 2.2. Kava Diversity

**Figure 3:**
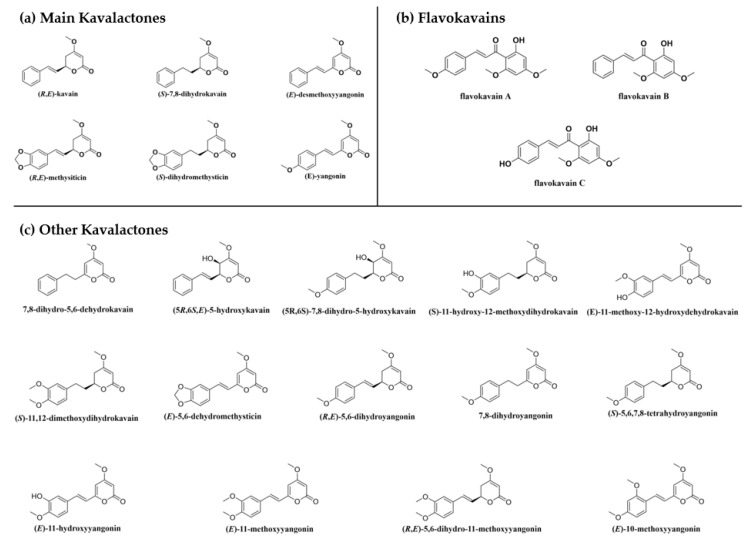
(**a**) Main kavalactones; (**b**) flavokavains; and (**c**) minor kavalactones in kava.

**Figure 4:**
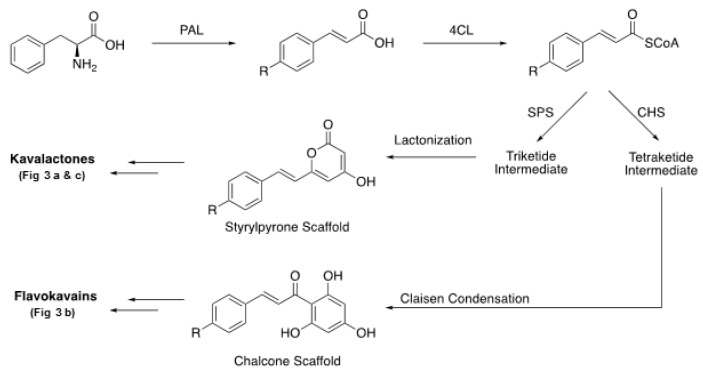
Biosynthetic pathways of kavalactones and flavokavains.

It is important to keep in mind that not all kava is equal in efficacy and safety and therefore it is critical to characterize a kava product with detailed knowledge of its preparation method and chemical composition. Many factors, ranging from cultivars, parts of the plant used, preparation methods, and solvents employed for extraction, affect the composition of the final kava products. The chemical components of kava with substantial reported biological activities are the kavalactones and flavokavains (Figure 3). Each class of compounds has unique properties aside from some overlaps in the reported biological effects. Most biological studies of kava have focused on the six major kavalactones (Figure 3a), which were reported to account for up to 20–50% of the dry weight of the kava root [32,33] and 96% of organic/lipid extracts [32]. Considering the importance of the kavalactones, it is not surprising that the critical factor contributing to the quality of kava comes from the relative concentrations of those six compounds. Flavokavains A, B and C are minor constituents even though they are generated through similar biosynthetic pathways as kavalactones (Figure 4) [34]. Given the recent reports of their biological activities (anticancer and hepatotoxic risk potentials), flavokavains may need to be included in quality control for kava products.

#### 2.2.1. Cultivars

**Figure 5:**
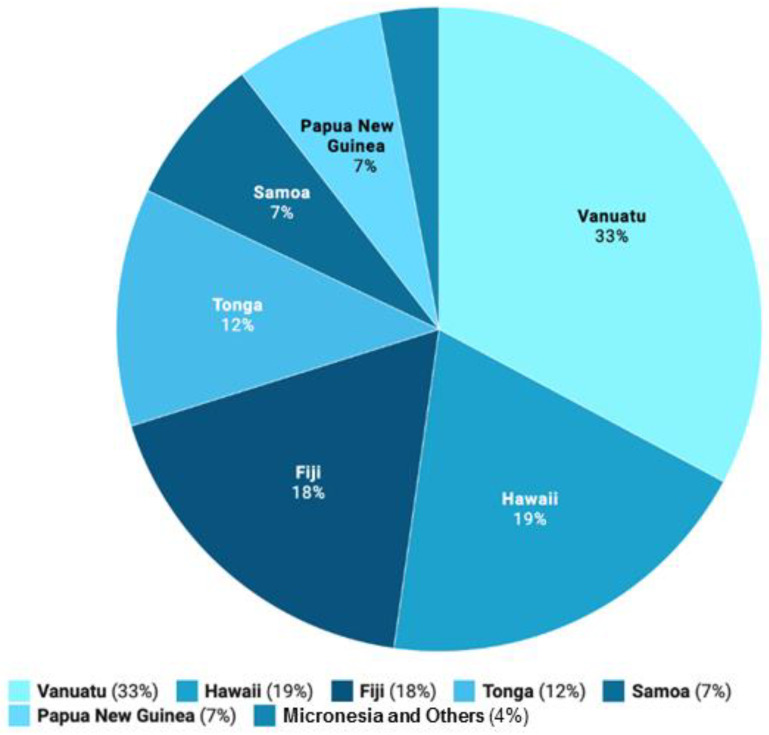
Distribution of kava cultivars among primary producers.

One main factor contributing to kava product variation is the cultivar type. There are over 150 different cultivars [35]. Different regions have different and often unique cultivars and/or chemotypes [36,37] (Figure 5). These different cultivars have varying levels of kavalactones and flavokavains [5,35,37,38]. Traditionally, chemotypes of kava are not discussed in terms of enumerated values of each kavalactone, but rather in terms of cultivar monikers such as “Melo Melo” and “Ronriki”. Since kavalactones are genetically controlled, the therapeutic properties of each cultivar are preserved through generations, and therefore this qualitative system of tracking kava is effective. In Vanuatu, kava cultivars are categorized into four groups, noble, medicinal, two-day (also known as tuidei), and wichmannii. Of these, only the cultivars classified as noble kava are permitted for export. The reason for this restriction is that the effects produced in the user vary widely, and noble cultivars produce a more desirable and safer experience. In particular, the presence of relatively high kavain content is associated with enhanced quality and efficacy of the anxiolytic properties that kava is best known for, while dihydromethysticin and dihydrokavain-forward cultivars produce more overpowering and/or unpredictable effects such as nausea and headaches that can last multiple days. Comparing kavalactones from noble cultivars to two-day cultivars further supports this, as noble kava contains kavain at the highest concentration, while tuidei kava contains significantly more dihydrokavain or dihydromethysticin. Flavokavains vary across cultivars as well. For example, the abundance of flavokavain A and B varies substantially among different kava cultivars, as characterized by Lebot et al. [39]. Noble cultivars were reported to have about one fourth the amount of flavokavains A and B relative to the two-day cultivars [39].

#### 2.2.2. Plant Parts

**Figure 6:**
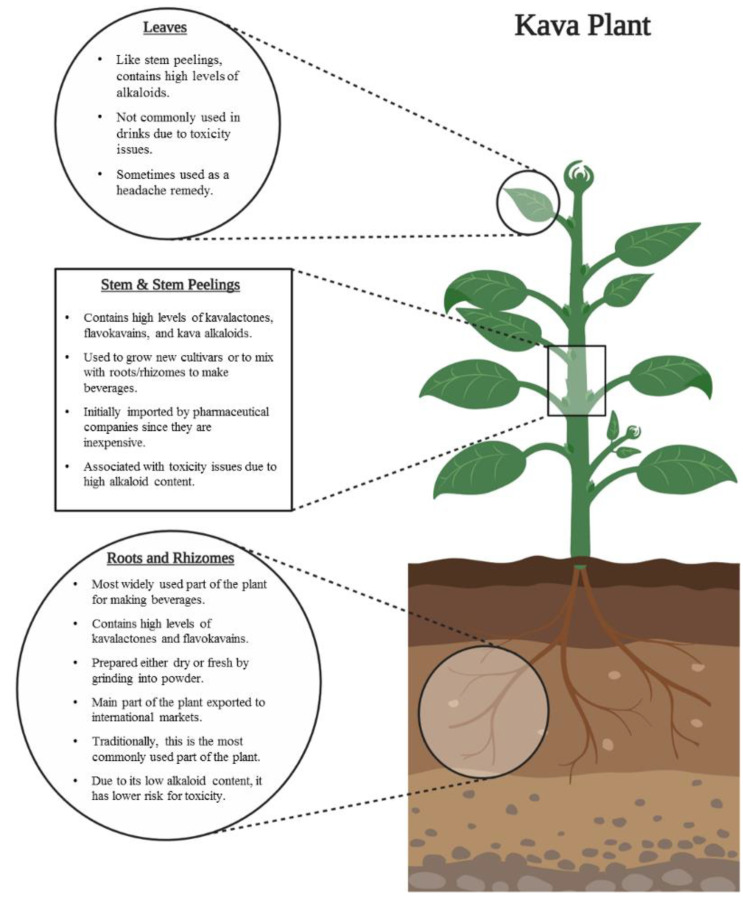
Different parts of kava plant and their features.

Kava can be prepared from different parts of the plant—the roots, the rhizomes and sometimes the stem peelings, but not the leaves (Figure 6). Although stem peelings have been employed to produce kava due to their high kavalactone content [35], they also have a higher content of alkaloids, which have been suggested as one of the potential reasons for the associated cytotoxicity [35].

#### 2.2.3. Extraction Solvents

Another major contributing factor to the different chemical compositions of kava products is the solvent used for extraction. The solvent for traditional kava preparations is water [15,36,38,40,41]. Since the introduction of kava consumption to Western society, other solvents have been used to optimize the kavalactone extraction yield, often resulting in higher concentration of these active compounds, but also other potentially less desirable (even “dangerous”, see later discussion) flavokavains and alkaloids [15,32,35,38,40,41]. For instance, the abundance of flavokavains A and B in the ethanolic kava preparation was reported to be ~100 times higher than in the traditional aqueous kava preparation [5]. In terms of yield, there seems to be a consensus that acetone is the best solvent for the extraction of kavalactone compounds [36,40,42,43], followed by ethanol, and finally water [36,40,42]. Many other factors also could contribute to the variations in kava composition, such as the age of the plant, the storage conditions, climate and soil, and potential adulterations.

### 2.3. Kava Standardization

Because of these variables, which could lead to different pharmacology and safety profiles, there is a crucial need for kava product standardization. The most common analytical method for kava characterization continues to be HPLC-based analysis [42]. This method is widely applied in industry and academia to fingerprint different cultivars, and to inform the actual kava dosage of the analyte product [42]. Other analytical methods include thin-layer chromatography (TLC), liquid chromatography–mass spectrometry (LC-MS), and UV-vis spectrometry.

With respect to chemotype determination, the method consists of assigning an ID number to the six major kavalactones according to their elution order in HPLC (Table 2) and analyzing their percent content [3,43,44]. This constitutes the product’s chemotype and is reported in decreasing order of concentration. Data on different kava chemotypes were adapted from Teschke and Lebot [42] and Noble and Tuidei assignment were referenced to the 2002 Kava Act [45]. The current method for kava product standardization is the sum of the six major kavalactones (Figure 3a) [42]. This standardization, however, has several limitations. First of all, it does not attempt to categorize the flavokavain content and other ingredients of the product. As reviewed later, the non-kavalactone ingredients may introduce new pharmacology or toxicity. In addition, the six major kavalactones could have different, and sometimes unique, pharmacological properties. Total kavalactone as a standardization is not expected to be sufficient. Detailed fingerprints of the individual kavalactones and other compositions may be necessary to better support future investigations and practice.

## 3. Regulation of the Inflammatory Responses by Kava and Its Components

Inflammation plays important roles in the pathology of a wide variety of diseases, ranging from cancer to neurological disorders [46]. Studies into kava and inflammation have been partly stimulated by its analgesic effects [47], as anti-inflammatory drugs are widely used for pain relief [48]. Historically, kava has been used for the treatment of urinary tract infections and immune-related disorders, such as asthma [49]. Some of the first robust demonstration of kava’s anti-inflammatory potential in a laboratory setting dated back to a study published in 1965. This study reported that natural kavalactones, particularly kavain, showed significant inhibitory effects on edema induced by either formalin, serotonin, carrageenan or dextran on rat paws, as well as on UV light-induced inflammation of the rat skin [50]. Multiple studies to date have evaluated the activities of kava and its isolated ingredients in various inflammation models, as well as analogs synthesized based on the structures of proposed active components, models, different dosages, different experimental conditions, and possibly different kava products (Table 3).

### 3.1. Effects of Kava Components on Inflammation

Kavain, dihydrokavain, methysticin, and dihydromethysticin (50 μg/mL) were investigated in the context of TNF-α production in human acute monocytic leukemia cells, as well as for their response to lipopolysaccharide (LPS)-induced lethality in lab animals [51]. All of these kavalactones were able to suppress LPS-induced TNF-α production, with kavain being the most potent. Kavain also protected C57 mice from lethal doses of LPS. In a model of Alzheimer’s disease, methysticin (6 mg/kg bodyweight) significantly reduced the secretion of TNF-α and IL-17A and improved the symptoms of the disease [52]. Methysticin was shown to activate the Nrf2 pathway in the mouse hippocampus and cortex [52], in which an antioxidant response was induced to maintain cellular redox homeostasis. This was consistent with the results from another study [53], which showed that methysticin, kavain, and yangonin activated the Nrf2 pathway in neuronal PC-12 and astroglial C6 cells. The effect of yangonin on estrogen-induced hepatic cholestasis, an inflammatory disease of the liver, was explored in C57BL/6 mice [54,55]. Yangonin treatment (20 mg/kg) reduced estrogen-induced cholestasis via activation of the Farnesoid X receptor (FXR). Desmethoxyyangonin (10 mg/kg) was also reported as an inhibitor of LPS-induced inflammation in murine macrophages and LPS/D-galactosamine (LPS/D-GaIN)-induced hepatitis in mice [56]. In this study, desmethoxyyangonin significantly inhibited the proliferation and activation of T cells and the activity of several pro-inflammatory mediators in vitro. Desmethoxyyangonin also protected against LPS/D-GalN−induced acute hepatic damage in mice (as indicated by decreased circulating aminotransferases) likely through the reduced infiltration of inflammatory macrophages, neutrophils and pathogenic T cells into liver tissues. In addition, desmethoxyyangonin pretreatment significantly improved the survival rate of LPS/D-GalN-treated mice. This study suggests that desmethoxyyangonin protected mice via regulating the IKK/NF-κB and Jak2/STAT3 inflammation signaling pathways [56].

In murine macrophages, flavokavains A, B and C reduced NO production stimulated by LPS [57]. A separate study observed a suppressive effect of flavokavain A on LPS-induced expression of pro-inflammatory mediators [58]. Specifically, flavokavain A (20 μM) suppressed the expression of the pro-inflammatory iNOS and COX-2, as well as the subsequent production of NO, PGE-2 and pro-inflammatory cytokines such as TNF-α, IL-1β and IL-6. The study found that these effects are mediated at least in part through inhibition of the JNK/p38 MAPK signaling pathways [58]. Furthermore, flavokavains A and B were shown to have immunomodulatory functions, such as the stimulation of splenocyte proliferation and the secretion of IL-2 and TNF-α in harvested splenocytes, as well as an increase in the subsets of T cell populations in BALB/c mice, with flavokavain B being more potent than flavokavain A (30 μg/mL) [59].

**Table 3:** Summary of inflammation-related effects and mechanisms.

Component Used	Inflammation-Related Pharmacological Effects	References
Whole kava extract	Upregulated microglial iNOS and serum IL-1β, IL-6, and TNF-α in Zebrafish Intracellular calcium influx and subsequent immune responses in mast cells	[60,61]
Yangonin	Ameliorated estrogen-induced cholestasis via Farnesoid X receptor signaling and improved inflammatory gene expression Suppression of pro-inflammatory NF-κB, TNF-α and IL-1β Activation of Nrf2 in neuronal PC-12 and astroglial C6 cells	[53,54,55]
Desmethoxyyangonin	Inhibition of LPS-induced inflammation studied in murine macrophages Inhibition of LPS/D-galactosamine-induced hepatitis in mice Inhibition of T cell activation and proliferation ex vivo and pro-inflammatory mediators in vitro Regulation of IKK/NF-κB and Jak2/STAT3 signaling pathways	[56]
Kavain	Activation of Nrf2 in neuronal PC-12 and astroglial C6 cells Immunization to lethal doses of LPS in C57 mice Suppression of LPS-induced TNF-α production Improved anti-inflammatory responses observed in several analogs Macrophage-dependent inflammatory hepatotoxicity in rat livers	[51,53,62,63,64,65]
Dihydrokavain	Suppression of LPS-induced TNF-α production	[51]
Methysticin	Suppression of LPS-induced TNF-α production Macrophage-dependent inflammatory hepatotoxicity in rat livers Reduced secretion TNF-α and IL-17A and Nrf2 activation in a model of Alzheimer’s disease Activation of Nrf2 in neuronal PC-12 and astroglial C6 cells	[51,52,53,65]
Dihydromethysticin	Suppression of LPS-induced TNF-α production	[51]
Flavokavains	Splenocyte proliferation, increased IL-2 and TNF-α secretion, reduced NO production in BALB/c mice Reduced LPS-induced NO production in murine macrophages Suppression of LPS-induced iNOS, COX-2 and PGE2 and inhibition of NF-κB and AP-1 signaling	[57,58,59]

### 3.2. Improving the Anti-Inflammatory Effects via Chemical Modifications

Several studies have optimized the anti-inflammatory potential of kava via medicinal structural modifications of kava compounds. One study evaluated the inhibitory effects of kavain analogs on P. gingivalis-induced inflammation in vitro and in vivo and found that Kava-205Me dose-dependently inhibited TNF-α secretion [63]. In the same study, Kava-205Me reduced the secretion of other cytokines such as IL-12, eotaxin, RANTES, IL-10 and IFN-γ in murine macrophages. Furthermore, Kava-205Me improved the rate of wound healing associated with soft tissue inflammation and osteoclast activation in a model of acute P. gingivalis-induced calvarial destruction, as well as inducing a reduction in paw swelling and joint destruction. Another kavain analog, namely Kava-241, decreased the number of inflammatory cells and osteoclasts within the joints of mice exposed to P. gingivalis, as well as decreasing serum TNF-α [62]. The potential of kavalactone analogs in reducing P. gingivalis-induced TNF-α production was also shown in macrophages [66]. Finally, a kavalactone analog, namely Compound 1, strongly inhibited LPS-stimulated iNOS induction and NO production, as well as Nrf2 signaling potentiation in vitro in microglial cells, highlighting the potential of kavalactone analogs to reduce neuroinflammation and maintain redox homeostasis [64]. An analog of flavokavains was observed to inhibit LPS-induced pro-inflammatory expression of iNOS, TNF-α, IL-1 β, and IL-6 in murine macrophages in a manner superior to flavokavains A and B [57].

Overall, kava and its ingredients have demonstrated their anti-inflammatory effects and their potential for ameliorating certain inflammation-related diseases. More systematic and detailed characterization of the individual components and their effects, however, is needed with respect to the active ingredients and mechanisms of action. It is important to note that inflammation-related symptoms have been reported in individuals who have consumed kava chronically, including dermal scaling in the form of ichthyotic dermatitis and delayed type hypersensitivity reactions [61]. Interestingly, although mast cells display robust intracellular calcium responses upon traditional kava exposure, with a concomitant release of pro-inflammatory mediators [61], these responses were not observed with treatments of individual or combinatorial kavalactones. These data suggest that water-soluble ingredients of kava, instead of kavalactones, may be responsible for the pro-inflammatory responses. Similarly, our recent study showed that, while an ethanolic kava extract stimulated intracellular calcium movements, this activity disappeared when the aqueous components were removed [67]. These results suggest that the aqueous components of kava extracts are responsible for the observed calcium movements, instead of the more hydrophobic ingredients, such as the kavalactones. Further in-depth studies may consider the removal of kava’s aqueous components.

## 4. Neurological Functions of Kava and Their Mechanisms

The psychoactive effects of kava use have been appreciated for a long time (Table 4). Its neurological benefits have made it a popular non-prescription treatment for multiple neurological disorders [11,68,69,70,71,72,73,74,75]. Kava use, however, is not free of side effects, such as headaches, fatigue, tremors and restlessness [76,77,78]. Most of the neuropharmacological properties of kava are attributed to kavalactones and a long list of putative targets have been proposed [79,80,81,82,83,84]. However, the detailed mechanisms are still poorly understood at the present.

### 4.1. Neuropharmacological Functions of Kava and Potential Mechanisms.

In one study, one week of kava administration (75 mg/kg) induced an increase in acetylcholinesterase (AchE) activity in the adult male rat striatum [85], while 4-week administration resulted in significant decreases in the enzymatic activity in the cortex, hippocampus, and striatum [85]. Another study found that kavain (up to 500 μM) reduced veratridine-induced intracellular calcium influx, glutamate release, and sodium channels in rat cerebrocortical synaptosomes, in a concentration-dependent manner [86]. Kavain, dihydrokavain, and yangonin were also found to inhibit human α1 glycine receptor activity in a concentration-dependent manner in HEK293 cells [87] while the binding of kavalactones and strychnine on the receptor was mutually exclusive [87]. Kava extract and individual kavalactones (25 μM) were shown to inhibit intracellular calcium influxes induced by norepinephrine treatment in lung cancer cells [67]. Sodium channel inhibition by methysticin and kavain was also observed in hippocampal neurons [82]. Kavalactones also inhibit human monoamine oxidase B (MAO-B) with low μM potency and, to a lesser extent, MAO-A [88]. A study showed that kavain induced a decrease in the extraneuronal 5-hydroxytryptamine receptor (5-HT, serotonin) [89]. The same study observed that low-dose kavain decreased accumbal dopamine, whereas high doses increased it. Such differential effects were also observed in other kavalactones. For instance, while yangonin treatment decreased dopamine, desmethoxyyangonin increased it, which is intriguing given their similar chemical structures (Figure 3a). Another study showed that kavain and dihydromethysticin may enhance the effects of 5HT1A agonist ipsapirone in the guinea pig hippocampus [90]. Both compounds also activated glutamatergic N-methyl-D-aspartate (NMDA) receptors and voltage-dependent calcium channels. Kavain, dihydrokavain, methysticin and dihydromethysticin also showed analgesic effects in mice when tested by the tail flick test and abdominal constriction methods [47]. Interestingly, naloxone, which can inhibit morphine-induced analgesia, was unable to reverse the antinociceptive activities of the kava extracts, suggesting a non-opiate pathway mechanism.

**Table 4:** Summary of neuropharmacological effects and mechanisms.

Component Used	Neuropharmacological Effects and Mechanisms	References
Whole kava extract	Enhancing of GABA_A_R binding by muscimol Increased EC_50_ of glycine at its receptor Non-opiate-related analgesic effects in mice	[47,87,91]
Yangonin	Dose-dependent inhibition of glycine receptor activity Decreased accumbal dopamine in mice Activation of Nrf2 pathway in neuronal and astroglial cells Affinity for human recombinant CB_1_ receptor, with selectivity vs. the CB_2_ receptor	[52,53,87,89,92]
Desmethoxyyangonin	Increased accumbal dopamine in mice Inhibition of p38/NF-κB/COX2 pathway activation	[89,93]
Kavain	Dose-dependent inhibition of veratridine-induced intracellular calcium influx, glutamate release, and sodium channels Dose-dependent inhibition of glycine receptor activity Activation of glutamatergic N-Methyl-D-aspartate (NMDA) receptors and voltage-dependent calcium channels in guinea pig hippocampus. Sodium channel inhibition in hippocampal neurons Decreased extraneuronal 5-HT Decreased accumbal dopamine at low doses, increase at high doses Enhancements of ipsapirone activity against 5HT_1A_ in hippocampus Non-opiate-related analgesic effects in mice Positive GABA_A_R modulation in *Xenopus* independent of benzodiazepine binding site Activation of Nrf2 pathway in neuronal and astroglial cells	[47,52,53,82,86,87,89,90,94]
Dihydrokavain	Dose-dependent inhibition of glycine receptor activity Non-opiate-related analgesic effects in mice	[47,87]
Methysticin	Sodium channel inhibition in hippocampal neurons Non-opiate-related analgesic effects in mice Activation of Nrf2 pathway in neuronal and astroglial cells	[47,52,53,82]
Dihydromethysticin	Enhancements of ipsapirone activity against 5HT_1A_ in hippocampus Activation of glutamatergic N-Methyl-D-aspartate (NMDA) receptors and voltage-dependent calcium channels in guinea pig hippocampus. Non-opiate-related analgesic effects in mice	[47,90]

A number of studies have explored the ability of kava components to interact with the GABA pathway. One study observed only weak activity on GABAA binding sites in rat brain synaptosomal membranes with weak effects on benzodiazepine binding [84]. Another study showed that kavain (300 μM) positively modulated human recombinant GABAA receptors expressed in Xenopus, with the greatest enhancement observed in the α4β2δ GABAAR receptor and, interestingly, no enhancement of GABAARs via the classical benzodiazepine binding site [94]. In yet another study, a kava extract enhanced the binding of the potent GABAAR agonist muscimol, with maximal potentiation in the hippocampus, followed by the amygdala and medulla oblongata, and such effects were re-capitulated by individual kavalactones [91].

Neuroprotective benefits have also been reported for kava and kavalactones [93]. An interesting observation is the ability of methysticin (6 mg/kg) to activate the anti-oxidative Nrf2 pathway in mouse hippocampi and cortex, leading to improvements in cognitive deficits in a mouse model of Alzheimer’s disease [52]. Methysticin, kavain and yangonin were also reported to activate this pathway in neuronal PC-12 and astroglial C6 cells with protection against amyloid-β peptide-induced neurotoxicity [53]. In terms of the endocannabinoid system, a structure–activity relationship (SAR) study showed that, among kavalactones, only yangonin exhibited binding for the human recombinant CB1 receptor, with selectivity over the CB2 receptor [92].

### 4.2. Clinical Evidence of Kava’s Neuropharmacological Effects

Kava is best known clinically for its anxiolytic activity, including its potential against generalized anxiety disorder (GAD), a prevalent and impairing disorder associated with extensive psychiatric and medical comorbidity and usually characterized by a chronic course. The Cochrane Review concluded that kava is superior to placebo and recommended it as a symptomatic treatment of anxiety (60–280 mg kavalactones/day) [24]. A number of studies have further shown that kava can be an alternative to benzodiazepines and selective serotonin re-uptake inhibitors (SSRIs), especially in patients with mild to moderate anxiety, as reviewed elsewhere [25,95,96,97]. Kava may therefore be a superior alternative to current anxiolytic agents, especially since its dependencies are seldom observed and its use have other benefits [14]. However, results from several recent studies are mixed. The most recent clinical trial of kava consumption for GAD patients, consisting of 171 participants undergoing 16-week kava use, observed no significant differences in anxiety reduction between kava consumption and placebo groups [98]. Anxiolytic activities were observed in a short-term trial [99]. One complicating factor is that different kava products were used in these trials.

In addition to anxiety, kava consumption may offer a variety of other psychopharmacological benefits. In the 1960s, kava was used clinically to treat epilepsy [74]. Studies by Steiner suggest that kava may reduce cravings associated with substances of abuse, such as alcohol, tobacco, cocaine, and heroin [100]. Kava has also been used as part of addiction rehabilitation programs in New Zealand with a reported 90% success rate [101]. Importantly, kava consumption appears to be clinically non-addictive [102]. Kava consumption has also been reported with improvements in recognition memory tasks [103] and enhanced accuracy and performance in visual attention and working memory tasks [14]. The anxiolytic and antidepressant activities were not associated with safety concerns [22]. Sleep-inducing and improving effects, with increased deep sleep periods and sleep spindle activity, have been reported as well [104]. Similar results were observed in patients with non-psychotic anxiety-related sleep disorders (200 mg kava/day) [105]. As sleep disorders are common in the general population, particularly in various neuroinflammatory and neuropsychiatric disorders, kava’s sleep-improving effects warrant further investigations in future research.

To date, most of the clinical trials have used on kava preparations. Different kava products, or at least products with different kavalactone compositions, have been used, which may account for some of the different clinical outcomes. A more rigorous characterization of kava composition is needed, and individual active ingredients should be evaluated in future clinical studies.

## 5. Kava and Cancer

Research on the potential anti-cancer activities of kava has been fueled by the epidemiological observations suggesting that kava consumption is associated with lower cancer incidence [106]. Nations with high kava consumptions, such as Vanuatu, Fiji and Western Samoa, have had much lower age-standardized cancer incidence rates in comparison to the rates in non-kava-drinking countries. Intriguingly, men in these regions had lower incidence rates of cancer than women, which is opposite to the general trends in other parts of the world; given that kava is mainly consumed by men, this observation is consistent with kava’s potential in reducing cancer risk.

### 5.1. Cancer Chemoprevention

#### 5.1.1. Lung Cancer

Kava and its components have demonstrated chemopreventive activity against carcinogen-induced tumorigenesis in several lab animal models (Table 5). Our group was the first to report that kava can inhibit lung tumorigenesis. Dietary supplementation of an ethanolic kava extract at a level of 10 mg/g showed a reduction in lung tumor multiplicity induced by 4-(methylnitrosamino)-1-(3-pyridyl)-1-butanone (NNK) plus benzo[a]pyrene (B[a]P) in A/J mice [107]. Mechanistically, the preventive efficacy was associated with the induction of apoptosis and the suppression of cell proliferation. In a later study [108], kava treatments, either in the diet at levels of 5, 2.5 and 1.25 mg/g or by daily gavage dosing covering the initiation stage, nearly completely blocked NNK-induced lung adenoma formation in A/J mice in association with a reduction in O6-methylguanine (O6-mG), a highly tumorigenic DNA damage induced by NNK in lung tissues. Interestingly, the kavalactone-enriched fraction fully recapitulated kava’s chemopreventive efficacy [108], supporting kavalactones as the active components. (+)-dihydromethysticin (DHM) was later identified as an active compound, which reduced 97% of adenoma multiplicity at a level of as low as 0.05 mg/g (50 ppm) in A/J mice [33], whereas (+)-dihydrokavain (DHK) was inactive [109]. Mechanistically, DHM-mediated reductions in DNA damage are independent of the aryl hydrocarbon receptor (AhR) [110]. DHM promoted the detoxification of 4-(methylnitrosamino)-1-(3-pyridyl)-1-butanol (NNAL, the major metabolite of NNK) by enhancing NNAL O-glucuronidation [111]. Moreover, DHM had a minimal effect on the activity of cytochrome P450 2A5 (CYP2A5), which catalyzes NNK and NNAL bioactivation in A/J mouse lungs.

In addition to these animal studies, we conducted a pilot clinical trial to evaluate the effects of kava on NNK metabolism in active smokers [112]. In this study, 21 smokers took kava capsules three times daily (standardized to 225 mg kavalactones per 3 capsules). Kava was shown to increase urinary excretion of total NNAL and reduce the levels of urinary 3-methyladenine, urinary total nicotine equivalents, plasma cortisol, and urinary total cortisol equivalents. These results support the potential of kava to reduce lung cancer risk among smokers.

#### 5.1.2. Other Cancers

Besides lung cancer, kava also showed preventive effects on urothelial cell carcinoma (UCC), prostate cancer, and colon cancer, likely through the inhibition of cell proliferation, induction of apoptosis and upregulation of cancer suppressor genes. Flavokavain A (6 g/kg food) inhibited the occurrence of high-grade UCC by 42.1% in association with the downregulation of Ki67, survivin, and the X-linked inhibitor of apoptotic proteins (XIAP) and the upregulation of p27 and DR5 in UPII-SV40T mice [113]. Dietary feeding of flavokavain A (3 g/kg diet) also inhibited prostate tumorigenesis and cancer progression in a transgenic adenocarcinoma of mouse prostate (TRAMP) mouse model by inducing apoptosis and anti-proliferation through the enhancement of S phase kinase-associated protein 2 (Skp2) degradation [114]. An ethanolic kava extract (6 g/kg diet) reduced dimethylhydrazine-induced colon cancer in a male Wistar rat model [115]. Our collaborative study of a diet with 0.4% kavalactone-enriched kava fraction B showed a decreased incidence of neuroendocrine carcinomas (NECa) and the suppressed growth of epithelial lesions in the TRAMP mouse model [116]. Such treatment suppressed a number of oncogenes related to angiogenesis and cell proliferation and upregulated tumor suppressor genes [116].

**Table 5:** Kava and its components in cancer prevention.

Components	Cancer Type	Mechanism	Model	Reference
Kava	Lung cancer	Reduction in NNK induced DNA damage	A/J mice	[107]
Kavalactone-rich fraction	Lung cancer	Reduction in NNK induced DNA damage	A/J mice	[108]
	Prostate cancer	Inhibition of angiogenesis and cell proliferation genes and upregulation of antitumor genes, immunity, muscle/neuro, and metabolism-related genes	Male C57BL/6J and female C57BL/6-Tg TRAMP 8247Ng/J mice	[116]
Nonpolar extract	Colon cancer	Reduction in precancerous lesions	Rat	[115]
DHM	Lung cancer	Reduction in NNK induced DNA damage and NNAL detoxification	A/J mice	[33,109,111]
	lung cancer	Reduction in NNK induced DNA damage	C57BL/6 Female Mice	[110]
Flavokavain A	Urothelial cancer	Induction of apoptosis	UPII-SV40T mice	[113]
	Prostate cancer	Inhibition of proliferation and induction of apoptosis	Female hemizygous C57BL/TGN TRAMP mice and male C57BL/6 mice	[114]

### 5.2. Anticancer Activities of Kava and Its Components 

Traditional kava, alone or combined with sea hibiscus, displayed anticancer activity against breast and colon cancer cells [117] and reduced prostate tumor growth in association with enhanced androgen receptor (AR) protein degradation in patient-derived prostate cancer xenograft models [118]. In the cell culture mode above, the unfiltered preparations were more active than the filtered samples [117]. Filtration reduced the content of kavalactones and chalcone-based flavokavains, suggesting that kavalactones and flavokavains are potentially anti-cancer components [117] (Table 6).

#### 5.2.1. Flavokavain A

Recent reports have documented the anticancer activity of chalcone-based flavokavain A in various cancer cell culture models. For example, flavokavain A was reported to inhibit cancer progression in taxol-resistant cancer cells [119], inhibit cancer cell proliferation and metastasis [120], and induce apoptosis [121], autophagy, and G2/M cell cycle arrest [22,122,123]. It was also reported to enhance antitumor immunity [124] and synergize with chemotherapies [125]. As another example, flavokavain A (30 μM) was reported to selectively inhibit cell proliferation and induce apoptosis in PTX-resistant A549/T cells by blocking the PI3K/Akt pathway, and reversing P-gp-mediated PTX resistance [119]. Flavokavain A was shown to induce G2/M arrest in a panel of cancer cell lines, including breast cancer cell lines (MDA-MB231 at 65 μM) [22], high-grade bladder cancer cell lines (T24, UMUC3, TCCSUP, 5637, HT1376, and HT1197 at 40 μM) [122] and prostate cancer cells (PC3 at 50 μM) [123]. Indeed, chalcone-based compounds have been well documented to induce cell cycle arrest at G2/M phase through inhibition of microtubule polymerization [126,127]. Flavokavain A also demonstrated anticancer activities in several lab animal models. It suppressed bladder cancer (T24) growth in a xenograft mouse model (50 mg/kg/d) [121]. At 200 mg/kg/d, flavokavain A inhibited lung metastasis of osteosarcoma cells in mice potentially via reducing Skp2 expression [120]. Lastly, flavokavain A was reported to enhance antitumor immunity and suppress the inflammatory process in breast cancer-challenged mice (50 mg/kg/d) [124].

#### 5.2.2. Flavokavain B

Flavokavain B was reported to have significant anti-tumor effects on several carcinoma cell lines both in vitro and in lab animal models. Similar to flavokavain A, flavokavain B was reported to inhibit cancer cell proliferation [118], metastasis [128,129] and angiogenesis [130], and induce apoptosis [128,129,131,132,133,134,135,136], G2/M [128,132,136,137], G0/G1 [138] cell cycle arrest, autophagy [134,139,140,141,142], and the immune responses [143]. Unlike flavokavain A, flavokavain B induced G2/M cell cycle arrest in cancer cells regardless of the p53 status [128,131,132,134,136,137], which is intriguing given their similar structures (Figure 2). Flavokavain B has also been reported to inhibit the uridine-cytidine kinase 2 (UCK2) enzyme in HT-29 cells, resulting in G0/G1 cell cycle arrest and subsequently led to cancer cell death through the MDM2-p53 signaling pathway (50 μM) [138]. Additionally, flavokavain B induced cell death through p21-mediated cell cycle arrest and the activation of p38MPAK in HeLa cells (17.5 μM) [144]. Other mechanisms have also been suggested for flavokavain B’s anticancer potential, but with limited data [128,129,130,143]. For example, Flavokavain B was shown to enhance the anticancer effect of bortezomib in PC3 and C4-2B cell lines, potentially via promoting Skp2 degradation in an ubiquitin and proteasome-dependent manner [145]. Flavokavain B enhanced the anticancer effects of daunorubicin (DNR) in DNR-resistant acute myeloid leukemia, potentially by modifying the NF-κB activation (10 μM) [146].

#### 5.2.3. Kavalactones

Several kavalactones were also reported to have anti-cancer potential [147,148,149]. For instance, DHM induced apoptosis in osteosarcoma cells through the modulation of the PI3K/Akt pathway, the disruption of mitochondrial membrane potential and the induction of cell cycle arrest at 25–100 μM [147]. It also suppressed the growth of colorectal cancer via the NLRC3/PI3K pathway at 25–100 μM [148]. Yangonin at 200 μM induced autophagy and sensitizes bladder cancer cells to flavokavain A and docetaxel via inhibition of the mTOR pathway [149]. Furthermore, kava extract (0–170 μg/mL) and several kavalactones (10–90 μM) showed inhibitory effects on the efflux transporter P-glycoprotein (P-gp) in P388 cells [150], which may be useful to overcome cancer drug resistance.

Although the anticancer properties of kava and its components have been reported in different types of cancer cell culture models, these data must be interpreted with caution. Firstly, the exposure concentrations used in many in vitro studies may have little physiological relevance because they are unlikely to achieve such concentrations in lab animals or in humans. For instance, in a self-medication study, the concentration of kavain in blood reached 40 ng/mL (about 0.17 μM) 1 h after an oral dose of 800 mg kavain [151], while the recommended daily dose of total kavalactones is typically less than 300 mg for human use. Secondly, many putative mechanisms have been reported with limited consensus except the G2/M phase cell-cycle arrest for flavokavains A and B, raising concerns of specificity. Given its α,β-unsaturated ketone functional groups, flavokavains A and B are labile to various nucleophiles via the Michael addition reaction, which may covalently modify a wide range of proteins [127]. This promiscuous nature may contribute to the reported multiple mechanisms of action. Lastly, flavokavain A and B are the most cytotoxic compounds in kava identified to date [152]. They have also been demonstrated to deplete glutathione [153] and cause hepatotoxicity in lab animals [153,154]. Their safety profile remains to be characterized, particularly given their high abundance in non-traditional supplement forms vs. low abundance in the traditional kava preparation. In summary, kava and some of its components have demonstrated chemopreventive and anticancer potential in cell culture models and a limited number of animal models. In depth pharmacokinetic and ADME studies of the individual kava phytochemicals and their combinations are necessary to establish the relevance of the “mechanisms” uncovered thus far and inform further research into the mechanisms in relevant in vitro models. Furthermore, more efficacy and safety testing in relevant animal cancer models will support and inform human clinical trials for cancer prevention or therapy uses.

**Table 6:** Kava and its components in cancer treatment.

Components	Cancer Type	Mechanism	Model	Reference
Water extract	Breast and colon cancer	Inhibition of proliferation	In vitro	[117]
kava root extract and flavokavain B	Prostate cancer	Downregulation of AR	Patient-derived prostate cancer xenografts in mice	[118]
Flavokavain A	Lung cancer	Anti-proliferation and induction of apoptosis, downregulation of P-gp	In vitro	[119]
	Breast cancer	Induction of apoptosis, inhibition of metastasis and G2/M cell cycle arrest	In vitro	[15]
		Inducing G2/M cell cycle arrest and enhancing the activity of Herceptin	In vitro	[125]
		enhancing antitumor immunity and inhibition of inflammation	Breast cancer-challenged mice	[124]
	Bladder cancer	Inducing G2/M cell cycle arrest	In vitro	[122]
		Induction of apoptosis and inhibition of proliferation	In vitro and xenograft mouse model	[121]
	Prostate cancer	Induction of G2/M cell cycle arrest and apoptosis and regulation of glutamine metabolism	In vitro	[123]
	Osteosarcoma	Inhibition of invasion through downregulation of SK	In vitro and osteosarcoma xenograft model	[120]
Flavokavain B	Lung cancer	Induction of G2/M cell cycle arrest and apoptosis	In vitro	[132]
		Induction of apoptosis and autophagy	In vitro	[139]
		Induction of apoptosis and inhibition of migration and invasion	In vitro	[129]
	Breast cancer	Induction of G2/M cell cycle arrest and inhibition of metastasis and angiogenesis	In vitro	[128]
		Induction of apoptosis and regulation of immune system	Xenograft model	[143]
		SAR study	In vitro	[137]
	Colon cancer	Induction of G2/M cell cycle arrest and apoptosis	In vitro	[134]
		Induction of G0/G1 cell cycle arrest	In vitro	[138]
	Gastric cancer	Induction of autophagy	AGS-xenografted mice	[140]
	Thyroid cancer	Inhibition of cell proliferation, migration and invasion and induction of apoptosis and autophagy	In vitro	[142]
	Oral cancer	Induction of G2/M cell cycle arrest and apoptosis	In vitro	[136]
	Squamous carcinoma	Inhibition of proliferation and induction of apoptosis and G2/M cell cycle arrest	In vitro and xenograft mouse model	[135]
	Synovial sarcomas	Induction of apoptosis	In vitro	[133]
	Glioblastoma multiforme	Induction of autophagy	In vitro and intracranial xenograft model	[141]
	Cervical cancer	Induction of p21-mediated cell cycle arrest	In vitro	[144]
	Osteosarcoma	Inhibition of cell proliferation and induction of apoptosis and G2/M cell cycle arrest	In vitro	[131]
	Brain endothelial cell	Inhibition of angiogenesis	In vitro and zebrafish	[130]
	Prostate cancer	Enhancing the activity of bortezomib through promoting Skp2 degradation	In vitro	[145]
		Downregulation of AR	Patient-derived prostate cancer xenograft model	[118]
	Acute myeloid leukemia	Induction of apoptosis and promoting the potency of daunorubicin via activation of NF-κB	In vitro	[146]
dihydromethysticin	Osteosarcoma	Induction of apoptosis and cell cycle arrest	In vitro	[147]
	Colorectal cancer	Inhibition of proliferation, migration, and invasion and induction of apoptosis and cell cycle arrest	In vitro and xenograft model	[148]
Yangonin	Bladder cancer	Induction of autophagy	In vitro	[149]
Crude extract and kavalactones	Mouse leukemia	Inhibition of P-gp	In vitro	[150]

## 6. Safety of Kava and Its Hepatotoxic Risk

Human exposure to kava differs significantly in terms of the dosing range, the dosing frequency and, more importantly, the methods of preparation and thus the chemical composition of kava products. These variations are expected to result in different safety profiles. Historically, there have been limited safety disputes surrounding traditional kava use [15]. Hepatotoxic risk has become a central topic since the early 2000s, due to a number of hepatotoxicity cases reported in Western countries [35,155,156,157,158,159,160,161,162,163,164,165]. These adverse events led to a ban on kava use as an herbal anxiolytic drug in Germany between 2002 and 2014 [27]. The U.S. Food and Drug Administration (FDA) also issued an advisory in March 2002. A number of causes have been hypothesized, including the potential use of low-quality cultivars, the adulteration of non-root parts of the plants, the improper handling and storage of kava materials, the potential for drug–herb interactions, kava overdosing, and others. However, none of these proposals have been validated because the product(s) associated with the purported cases have never been accurately documented. Kava’s hepatotoxic potential has therefore also been proposed as an idiosyncratic phenomenon [15]. With these challenges in mind, this section mainly focuses on kava’s safety data in relation to human exposure. A few lab animal results published during the past 15 years are discussed to cross-inform potential mechanisms of such toxicity issues. Results using biochemical and in vitro models are not reiterated herein because they have been covered in a number of reviews, including two recent ones [68,157].

### 6.1. Kava Safety in Lab Animals

Our group evaluated the potential benefits and risks of kava use since 2008. The safety of an ethanolic extract of kava from Gaia Herbs was monitored in several animal models with kava’s cancer prevention potential as the primary research focus [107,108,115,154,166]. In all of these studies, ethanol and water solvent residues in the kava product were removed. The abundance of the six major kavalactones accounts for about 50% of the dry mass of this ethanolic extract [33]. Kava was administered to mice or rats in their diet at a level of 1.25–10 mg/g with an exposure period between 14–30 weeks [107,115,166]. Kava’s safety profile was monitored by bodyweight, liver weight, relative liver weight, serum ALT, AST, GGT or liver pathology with no signs of adverse effects. Similar results were observed when kava was evaluated in C57BL/6 female mice for 14 weeks at a dose of 500 mg/kg of bodyweight via daily oral gavage, gauged by serum ALT and AST [154]. Kava’s hepatotoxic risk has also been speculated to be associated with potential drug–herb interactions [157,167]. Kava and its ingredients have been reported to modulate drug-metabolizing enzymes, particularly CYP enzymes, making drug–herb interactions mechanistically plausible. Since no signs of liver toxicity were observed in three different strains of rodents with kava dosages significantly higher than human exposure, we also explored the potential interaction of kava with the liver toxin drug acetaminophen (Tylenol) as a model compound. Surprisingly, even three days of kava pre-exposure greatly enhanced the hepatotoxicity of acetaminophen in C57BL/6 mice [154]. Through fractionation, this model identified flavokavains A and B as the potential ingredients for enhancing liver toxicity of acetaminophen. Nonetheless, the clinical relevance of these lab animal results remains an open question because acetaminophen exposure was documented in only one of the kava hepatotoxicity cases. It also remains to be determined whether kava enhances the hepatotoxic risk of other liver toxins, such as alcohol consumption, which may be more clinically relevant based on recent trial results from Sarris et al. [98].

The most comprehensive lab animal safety evaluation of kava was performed by the U.S. National Toxicology Program (NTP) [168]. Both genders of F344/N rats and B6C3F1 mice were evaluated with 5-day weekly kava gavage exposure for 2 weeks, 3 months, and 2 years. In these studies, “Kava Kava Extract” was obtained from Cosmopolitan Trading Co (Seattle, WA, USA). Interestingly, the product was described as a medium yellow powder at a density of 0.46 g/mL. No detailed preparation procedures could be found in the report about this kava product. Based on the description of the product, the “Kava Kava Extract” was likely ground kava powder instead of an extract form, because ethanolic extracts, like the one from Gaia Herbs, are an oil form of high viscosity and the acetonic extract is expected to have similar physical properties. If the product used in these NTP studies was indeed kava root powder, the results need to be interpreted again with caution because the powder, administered whole and directly to the rodents, is substantially different from the traditional and anxiolytic kava forms that humans use. The powder would contain all chemicals in kava, particularly those hydrophobic compounds, such as flavokavains A and B, that are of low abundance in the traditional form of kava. The pharmacokinetics in the powder matrix are also expected to be substantially different. Nonetheless, in the 2-week study, kava was given at dosages of 0, 0.125, 0.25, 0.5, 1.0, or 2.0 g/kg of bodyweight for both mice and rats. Increased liver weight was observed when the kava dose was 0.5 g/kg of bodyweight or higher. For the 3-month study, kava dosages were the same as the 2-week study and a liver weight increase was observed when the kava dose was 0.25 g/kg or higher. In the 2-year study, kava doses were 0, 0.1, 0.3, or 1.0 g/kg of bodyweight. Changes in liver weight were observed at a dose of 0.3 g/kg or higher. Pathological lesions in livers were only observed when the kava dose was 1.0 g/kg in rats. In short, the dosages of kava affecting liver weight and pathology in these studies are unlikely to be physiologically relevant as a life-long consumption of 4–5 g of neat kava root daily would be required for a human of 75 kg bodyweight. A few follow-up studies further analyzed the liver tissues from these studies, including immunohistochemical analyses of several CYP enzymes [169] and global gene expression [170,171]. No significant changes were observed when the kava dose was 0.3 g/kg or lower.

Based on these long-term exposure results, kava appeared to be well tolerated in rodents, particularly at a dosage not significantly higher than the human equivalent exposure. However, the products used in these studies are not well characterized, including our own studies, which need to improve in the future. In addition, there have been no lab animal safety studies with aqueous kava preparations, which more closely mimic the traditional kava formula.

### 6.2. Safety Data of Traditional Kava

The amount of kavalactone intake among traditional kava users is typically >750 mg/day [15,16,17,18], significantly higher than its anxiolytic and dietary supplement dose. In the context of the hepatotoxic risk associated with kava, Lee commented that “the Pohnpean dose is 8 times the recommended daily dose. If kava were toxic in its water-extracted form, we would expect to see an epidemic of hepatotoxicity in Pohnpei and other regions. However, this does not seem to be the case, based on my experience as a medical doctor in Micronesia, where kava is commonly consumed” [17]. Traditional kava use, therefore, is well accepted to be safe, which is in alignment with the recommendation from the WHO in its 2016 report [15]. Given the significant dose difference between traditional kava and kava as an anxiolytic agent or a dietary supplement, a potential solution to kava’s safety is to prepare it with its traditional composition or use a preparation method that removes the harmful components.

### 6.3. Safety of Kava as an Herbal Anxiolytic Drug

Between the late 1990s and early 2000s, over 100 hepatotoxic cases were reported to be associated with kava exposure, with the majority of cases in which kava was used as an anxiolytic agent. Most of these purported cases, however, were not well documented, and there is limited evidence to support a causal relationship [172]. Even if all of these cases resulted from kava use, the estimated risk would be less than 0.3 cases per one million daily doses [167,173], which is still superior to some, if not all, commonly used anxiolytic agents. At the same time, stress and anxiety are becoming more prevalent among the general population [174], while current pharmacologic treatments have only a modest clinical effect and carry a high burden of side effects [175,176]. Thus, there is an unmet clinical need for novel anxiolytics to achieve better anxiety management with minimal adverse effects [177]. Given its relaxing properties and questionable hepatotoxic risk, kava may reemerge as an anxiolytic drug in the future, which is further supported by the recent ruling in the German court [27]. It is therefore important to review the hepatotoxic cases associated with kava’s anxiolytic use, particularly those cases that might have a potential causal relationship. Knowledge from those incidents may provide insights into potential causes and identify clinical parameters for future safety monitoring. Among various evaluations of kava’s hepatotoxic risks, Teschke et al. employed the scale of the Council for International Organizations of Medical Sciences (CIOMS) to review the reported hepatotoxic cases [172]. A total of 14 cases were identified with possible or probable causality for kava. Some of the pertinent information is summarized in Table 7. The dose and period of kava use varied dramatically among these cases. Nine cases involved the use of other medications or dietary supplements, and two cases documented the use of aqueous kava extracts. Twelve cases were females, suggesting a higher risk in females compared to males. None of these cases had detailed descriptions about the kava products.

### 6.4. Safety of Kava in Recent Clinical Trials

There has only been a limited number of kava clinical trials in the past 15 years due its hepatotoxic risk. A few of these trials were reported by Sarris et al. [23,98,178], which evaluated aqueous preparations of kava for its anxiolytic potential among different populations. In an earlier one-week, three-arm trial [178], an aqueous kava product from a specific cultivar (Palarasul) was manufactured by MediHerbPty Ltd. (Warwick, Australia) and standardized to 60 mg kavalactones. This kava product was given to individuals of mild to moderate anxiety for one week with no changes in liver enzymes. The product was described qualitatively to have higher concentrations of dihydrokavain, kavain, and dihydromethysticin, a moderate level of methysticin, and lower levels of yangonin and desmethoxyyangonin with no information on other components. In a later 6-week randomized trial [23], Sarris used an aqueous kava product at a dose of 120–240 mg kavalactones daily and reported no changes in serum liver enzymes during the whole course of the trial. The kava tablets used in this trial were prepared by Integria Healthcare and independently characterized by Southern Cross University to have higher concentrations of dihydrokavain, kavain, and dihydromethysticin, moderate levels of methysticin and yangonin, and a lower level of desmethoxyyangonin in a qualitative manner. In a recent double-blind randomized placebo controlled trial [98], a new kava aqueous extract was prepared by Integria Healthcare, specifically for this trial, with a noble kava cultivar. This product was given to GAD patients for 16 weeks at a dose of 120 mg kavalactones daily. As early as 2 weeks, more frequent elevations of serum liver enzymes were observed in the kava treatment group. The participants in the kava group were also reported to have a higher incidence of alcohol use [98]. The authors quantitatively reported the composition of the kava preparation: kavain (29.23%), dihydrokavain (24.42%), trans-yangonin (13.88%), cis-yangonin (0.66%), desmethoxyyangonin (10.21%), dihydromethysticin (10.31%), and methysticin (11.30%). Surprisingly, a substantial amount of flavokavains A and B (4.35%) was reported in this kava formula, which appeared to be different from the other reports about aqueous kava preparations [5]. It should be noted that the three trials by Sarris used three different kava preparations. Whether and to what extent the different outcomes are due to different kava compositions remains an open question. Without rigorous characterization of the kava products, we will continue to face the same dilemma—is kava bad or are there bad types of kava?

We recently completed a one-week pilot kava trial among active smokers to investigate its potential in reducing tobacco smoke-associated lung carcinogenesis risk [112]. Participants were asked to refrain from acetaminophen and alcohol consumption and consumed a soft gel capsule of an ethanolic kava extract from Gaia Herbs at a dose of 225 mg total kavalactones. One-week exposure showed no adverse effects [112]. Kavain, dihydrokavain, methysticin, dihydromethysticin, and desmethoxyyangonin were quantified to account for 33.7%, 18.2%, 10.4%, 27.3% and 10.4%, respectively, of the total kavalactones [179].

### 6.5. Kava as a Dietary Supplement in the U.S.

Kava has been marketed as a dietary supplement in the U.S. to help manage stress and improve relaxation for several decades, with a wide range of products on the market from multiple suppliers. Many of the products are in the form of capsules filled with the ground powders of kava root. This is a potential problem because consumers would likely take the capsules as a whole, which exposes them to all of the chemicals in the root. Such a scenario is different from kava’s traditional use and may change its safety profiles. Some products are filled with dried concentrated ethanolic extracts in the form of capsules. Some kava dietary supplements are in the form of tinctures containing 95% ethanol. This form could be risky given kava’s potential to enhance the hepatotoxicity of liver toxins, particularly given the recent clinical results [98]. Currently, there are no published data about the amount of kava being consumed in any of these formats. Nonetheless, based on the export data from Fiji alone [5], the amount of kava imported into the U.S. each year is expected to be sufficient for 45 million doses of the daily recommended amount. Based on the CFSAN Adverse Event Reporting System (CAERS) database, there were a total of 30 adverse event reports between 2004 and 2019 that were potentially associated with kava exposure with no or limited product information. Although these data are not rigorous, they provide valuable information about kava exposure and risks; the reported adverse events associated with kava use are no more than 0.045 cases per million kava daily doses.

### 6.6. Safety Issues of Kava with No or Limited Knowledge of Composition

During the past 15 years, there have been a number of case reports in the literature about the potential adverse effects of kava where the product used was not reported or characterized. Berry et al. reported four cases of drivers in Iowa unable to safely operate a motor vehicle, potentially due to kava use, between 2011 and 2018 [180]. There was no detailed information on the kava products or dosages (the amount of kavalactones) used. Kava exposure was confirmed in two of the four cases based on the detection of yangonin and methysticin in the urine. On the other hand, because of kava’s potential in modulating the CNS, the influence of kava on driving has been evaluated in a number of clinical trials [181,182,183]. In a study by Herberg et al., a WS 1490 kava preparation was used at a dose of 300 mg kavalactone daily for 8 days [181]. A single dose of kava containing 180 mg kavalactones was used by Sarris et al. [183], while kava root, at a dose of 1 g/kg of bodyweight, was used in a study by Foo et al. [182]. No impairments were detected in any of these trials with a range of different tests.

On the other hand, a few cases of liver issues associated with traditional kava use have been reported [184]. Christl et al. reported a 42-year-old white male to have liver problems around 20 days after returning from Samoa, where he self-reported to consume 1–2 L in total of traditional kava [184]. The patient did not report alcohol overuse or the consumption of other medications. Laboratory tests showed markedly elevated liver enzymes (AST, 1,602 U/L; ALT, 2,841 U/L; ALP, 285 U/L), lactate dehydrogenase (460 U/L), and bilirubin (9.3 mg/dL). No information was reported about the kava product. Becker et al. also reported a patient with liver injury potentially associated with kava use where liver symptoms presented after 52 days of using a kava product with the detection of kavalactones and flavocaine B (potentially flavokavain B) [185]. However, many other compounds not reported to be contained in kava were also detected in the product consumed, suggesting that the product may have been adulterated [185]. Ketola et al. reported a suicidal intravenous injection of kava in combination with ethanol [186]. Although the cause of death could not be firmly established, all six major kavalactones were detected in the urine and blood with their concentrations in the range of 0.5–10 µM in the blood. Concentrations much higher than these have been extensively used in biochemical and in vitro assays to interrogate the mechanisms of action and various biological activities that may not be physiologically relevant.

### 6.7. Safety of Individual Kava Compounds

In comparison to kava, there has been no reported human exposure to pure compounds from kava in recent years. There are a few lab animal safety studies, which are briefly summarized in this section.

Yangonin was recently reported to protect against high-fat induced non-alcoholic fatty liver disease through the FXR in C57BL/6 mice [187]. Desmethoxyyangonin was also reported to protect against LPS/D-GalN-induced acute hepatic damage in mice [56]. Given the structural similarities among kavalactones, systematic investigations are needed to fully appreciate the potential of kavalactones in liver protection. A collaborative study between Lu and our group evaluated the potential of a kavalactone-rich kava preparation against prostate carcinogenesis in diet at a level of 4 mg/g [116]. The exposure started from 8 weeks of age to 16 or 28 weeks of age. There were no changes in liver enzymes, including ALT, AST, ALP and total bilirubin. We also evaluated the most lung cancer chemopreventive kavalactone dihydromethysticin at a level of 0.5 mg/g of diet for 8 and 17 weeks in A/J mice [33] with no signs of liver toxicity.

A number of studies report that flavokavains A and B are the most cytotoxic compounds identified in kava [152], and one study particularly demonstrated its toxicity towards cells of liver origin and showed that they increased the cellular level of AST, indicative of potential liver toxicity [188]. We showed that flavokavain A and B enhanced the hepatotoxicity of acetaminophen, reflected by the elevation of serum liver enzyme levels and more severe liver damage pathology [154]. Flavokavain B alone was also reported to induce hepatotoxicity in an animal model at a human exposure-relevant dose [153]. Because of the inherent α,β-unsaturated ketone functional group [127], flavokavains A and B are expected to react with various nucleophiles. Flavokavain B was indeed reported to deplete glutathione [153], which may lower the liver’s ability to defend against oxidative damage and increase hepatotoxic risk. Lastly, cultivars with a higher content of flavokavains A and B (non-noble cultivars) have a much shorter maturation time and provide higher yields, resulting in the promotion of growing such cultivars during the late 1990s due to the high demand for kava. The products from these cultivars reached the market before the onset of the purported hepatotoxic cases associated with kava use [157]. These, overall, suggest that flavokavains A and B in some kava preparations deviate in abundance from the traditional kava preparations and may account for the purported hepatotoxicity risk associated with kava use. On the other hand, Zi et al. have evaluated the safety of flavokavain A at a dose of 6 mg/g in FVB/N mice for three weeks [189] with no signs of hepatotoxic risk. Nonetheless, the authors noted increases in phase II enzymes and an increase in liver/body weight ratio, which suggest liver metabolic adaption to this compound and needs further characterization in relation to dose–response.

The evidence overall suggests that kava’s hepatotoxic risk is very low. There are a number of factors that may contribute to such a risk, which can be reduced if not eliminated. Given that not all kava products are the same, we submit that rigorous characterization and QC of kava products in future studies is one key prerequisite for our better understanding of kava’s safety and efficacy.

## 7. Conclusions and Future Directions

Overall, designer kava preparations could be promising clinical herbal modalities, given the totality of analyses of the biological activities and safety profiles of the different forms, fractions and individual compounds. The dietary supplement status of kava extracts in the U.S. also positions them well as clinical nutrients. However, to realize kava’s full potential for health promotion and for clinical indications, we must address several challenges.

First and foremost, we must strive to address the lack of rigorous standardization in its cultivation, harvest and post-harvest handling, extraction, fractionation and manufacturing for commercialization, preclinical animal investigation, and human clinical evaluations. The lack of standardization, at least in part, caused many contradictory observations that were difficult to reconcile and raised concerns about product safety.

Second, we must strive to fill the critical knowledge gaps of kava’s foundational pharmacology, pharmacokinetics, and ADME. Different compounds in kava have different bioactivities and mechanisms of action, and these compounds may interact with each other both pharmacokinetically and pharmacodynamically. There is very limited pharmacokinetic information. More systematic, detailed PK and metabolism studies are needed to establish the ADME of kava compounds and to define the relevant achievable concentration ranges after physiological and pharmacological exposure. Such information will guide the design of in vitro and ex vivo models with relevant exposure concentrations and will assist animal models to reveal relevant molecular targets and cellular mechanisms of the individual components of kava ingredients.

Finally, we should creatively design and conduct early stage human clinical trials to inform the optimal design and execution of future randomized controlled trials for the efficacy and safety of designer kava modalities for specific disease indications.

## Figures and Tables

**Table 1:** Reported dose of kavalactones in traditional kava use.

Major Regions	Traditional Kava Dose	References
**Vanuatu**	Female: 750 mg/day; male: 1000 mg/day	[15]
**Fiji**	>8000 mg/drink	[16]
**Tonga**	2400 mg/day	[17]
**Hawaii**	1000–1500 mg/drink	[18]

**Table 2:** Chemotypes and kavalactone quantities of noble and tuidei kava cultivars.

	Noble							
	**Puariki**	**Kelai**	**Borogu**	**Borogu**	**Borogu**	**Borogu**	**Borogu**	**Borogu**
DMY 1 (%)	0.71	0.91	0.74	0.84	0.7	0.51	0.67	0.61
DHK 2 (%)	1.49	2.21	2.33	2.16	2.29	2.05	1.94	1.44
Y 3 (%)	0.99	1.57	2.06	1.94	1.88	1.74	1.69	1.34
K 4 (%)	2.7	4.05	3.26	3.53	3.21	2.74	2.88	2.42
DHM 5 (%)	0.63	0.71	0.95	0.96	0.96	0.95	0.82	0.74
M 6 (%)	0.43	0.64	0.71	0.86	0.79	0.69	0.71	0.69
Total (%)	6.95	10.09	10.05	10.29	9.83	8.68	8.71	7.24
Chemotype	423156	423156	423516	423561	423561	423561	423561	423561
	**Borogu**	**Borogu**	**Borogu**	**Borogu**	**Borogu**	**Borogu**	**Silese**	**Ahouia**
DMY 1 (%)	0.78	0.38	0.47	0.46	0.43	0.45	0.64	0.78
DHK 2 (%)	2.17	1.12	1.48	1.17	1.24	1.47	1.5	1.83
Y 3 (%)	1.91	0.74	0.88	0.84	0.8	0.7	1.43	1.84
K 4 (%)	3.73	1.92	2.35	2.38	2.01	2.23	2.69	3.27
DHM 5 (%)	1.19	0.66	0.72	0.48	0.69	0.57	0.75	0.86
M 6 (%)	1.13	0.63	0.67	0.67	0.66	0.6	0.75	0.73
Total (%)	10.91	5.45	6.57	6	5.83	6.02	7.76	9.31
Chemotype	423561	423561	423561	423651	423561	423651	423651	432516
	**Tuidei**							
	**Tarivoravora**	**Abogae**	**Gelav**	**Marino**	**Rongrongwul**	**PD Palisi**	**PA Palisi**	**PC Palisi**
DMY 1 (%)	0.71	0.61	0.8	0.63	0.84	0.59	0.57	0.41
DHK 2 (%)	2.7	2.63	3.03	2.46	4.04	2.23	1.95	2.24
Y 3 (%)	1.66	1.35	1.98	1.49	1.62	1.41	1.43	1.29
K 4 (%)	2.46	1.89	2.7	1.93	2.65	1.83	1.74	1.69
DHM 5 (%)	1.78	1.77	2.07	1.71	2.44	1.77	1.71	1.52
M 6 (%)	1.42	1.15	1.46	0.96	1.32	1.39	1.39	1.3
Total (%)	10.73	9.4	12.04	9.18	12.91	9.22	8.79	8.45
Chemotype	245361	245361	245361	245361	245361	245361	245361	245631
	**Narango Palisi**	**Sara Palisi**	**Tarivarus**	**Malogro**	**Ring**			
DMY 1 (%)	0.48	0.57	0.91	0.36	0.81			
DHK 2 (%)	2.12	2.06	5.21	2.17	0.1			
Y 3 (%)	1.31	1.1	1.86	1.24	0.45			
K 4 (%)	1.62	1.91	2.6	1.6	0.13			
DHM 5 (%)	1.74	1.93	3.09	1.66	1.98			
M 6 (%)	1.45	1.46	1.31	1.38	0.37			
Total (%)	8.72	9.03	14.98	8.41	3.84			
Chemotype	254631	254631	254361	254631	521364			

**Table 7:** Clinical data of cases of causality relationship with kava exposure [172].

Patient Identification	Specific Information
BfArm 93015209 38 years Female	Acetonic kava extract (210 mg/day, 3.5 m). Oral contraceptive, Diazepam, and L-Thyroxine ALT 2305 U/L, AST 1048 U/L and ALP 307 U/L
BfArm 01006939 36 years Male	Acetonic kava extract (70 mg/d, 1.5 m) ALT 2341 U/L, AST 2425 U/L, and ALP 530 U/L
BfArM 01010536 45 years Female	Ethanolic kava extract (45 mg/d, 4 m) ALT 1000 U/L, AST 700 U/L, and ALP 360 U/L
BfArM 02001414 46 years Female	Ethanolic kava extract (360 mg/d, 1 m) ALT 1442 U/L, AST 683 U/L, and ALP 325 U/L
BrArM 02002090 26 years Female	Ethanolic kava extract (50 mg/d, 0.25 m) Sulfasalazine, Diclofenac, Progesterone, Omeprazole, Butylscopolaminium bromide ALT 572 U/L, AST 220 U/L, and ALP 163 U/L
BfArM 39 years Female	Ethanolic kava extract (60 mg/d, 6 m) Oral contraceptive, Paroxetine, and St. John’s wort ALT 600 U/L, AST 400 U/L, and ALP 183 U/L
BfArM 60 years Female	Ethanolic kava extract (1200 mg/d, 12 m) Etilefrine and Pretanide ALT > 1000 U/L, AST > 1000 U/L, and ALP > 500 U/L
IKS 2000–3502 50 years Male	Acetonic kava extract (280 mg/d, 2 m) ALT max 3627 U/L, AST max 3360 U/L, and ALP max 430 U/L
IKS 1999–2596 46 years Female	Acetonic kava extract (140 mg/d, 3m) Hydrochlorothiazine, Valsartan, and Propranolol ALT max 1900 U/L, AST max 2005 U/L, and ALP not recorded
59 years Female	Aqueous kava extract (unknown daily dose, 1 m) Lisinopril, Phenobarbital and Fenofibrate ALT 568 U/L, AST 672 U/L, and ALP not recorded
55 years Female	Aqueous kava extract (2.571 mg/d, 1.25 m) ALT 1666 U/L, AST 1569 U/L and ALP not recorded
56 years Female	Kava mixture (180 mg/d, 3 m) Passiflora incarnate, Scutellaria lateriflora, vitamins, and mineral supplements ALT 4539 U/L, ALP 190 U/L, and AST not recorded
14 years Female	Kava mixture (200 mg/d, 4 m) St. John’s wort, catnip root, Siberian ginseng root, and other 20 ingredients ALT > 4400 U/L, AST > 3500 U/L and AST not recorded
34 years Female	Aqueous powdered kava extract, ethanolic extract before (120 mg/d, 3 m) L-Thyroxine and potassium iodine ALT 884 U/L, AST 547 U/L, and ALP 319 U/L

## Abbreviations

WHO	World Health Organization
GC-MS	Gas Chromatography Mass Spectrometry
HPLC	High-performance liquid chromatography
TLC	Thin-Layer Chromatography
LC-MS	Liquid Chromatography–Mass Spectrometry
ROS	Reactive Oxygen Species
IL	Interleukin
TNF	Tumor Necrosis Factor
LPS	Lipopolysaccharide
NOS	Nitric Oxide Synthase
COX-2	Cyclooxygenase 2
NO	Nitric Oxide
PGE-2	Prostaglandin E2
NF-κB	Nuclear Factor kappa B
AP-1	Activator Protein 1
JNK	c-Jun N-terminal Kinase
MAPK	Mitogen-Activated Protein Kinase
FXR	Farnesoid X Receptor
Nrf2	Nuclear Factor Erythroid 2-related Factor 2
D-GaIN	D-galactosamine
AchE	Acetylcholinesterase
MAO	Monoamine oxidase
5-HT	Serotonin
NMDA	N-Methyl-D-aspartate
GABA	γ-aminobutyric Acid
GAD	Generalized Anxiety Disorder
SSRI	Selective Serotonin Reuptake Inhibitor
NNK	4-(methylnitrosamino)-1-(3-pyridyl)-1-butanone
B[a]P	Benzo[a]pyrene
DHM	Dihydromethysticin
DHK	Dihydrokavain
AhR	Aryl Hydrocarbon Receptor
NNAL	4-(methylnitrosamino)-1-(3-pyridyl)-1-butanol
CYP2A5	Cytochrome P450 2A5
UCC	Urothelial cell carcinoma
TRAMP	Transgenic Adenocarcinoma of Mouse Prostate
Skp2	S phase Kinase-associated Protein 2
NECa	Neuroendocrine Carcinomas
AR	Androgen Receptor
NSCLC	Non-Small Cell Lung Cancer
UCK2	Uridine-Cytidine Kinase 2
XIAP	X-Linked Inhibitor of Apoptosis
FDA	Food and Drug Administration
ALT	Alanine Transaminase
AST	Aspartate transaminase
GGT	Gamma-glutamyltransferase
NTP	National Toxicology Program
BZD	Benzodiazepine
CIOMS	Council for International Organizations of Medical Sciences
ALP	Alkaline phosphatase
CAERS	CFSAN Adverse Event Reporting System
ADME	Absorption, distribution, metabolism, and excretion
CFSAN	Center for Food Safety and Applied Nutrition
QC	Quality control
